# A multicenter, prospective, non-interventional drug intensive monitoring study of olaparib in a large real-world Chinese patient cohort with ovarian cancer (DIM-OC)

**DOI:** 10.1186/s13048-025-01924-8

**Published:** 2025-12-17

**Authors:** Li Wang, Lingling Xie, Qingshui Li, Yang Shen, Huaying Wang, Li Sun, Hongying Yang, Dongyan Cao, Haiying Li, Bei Lin, Qionghua Chen, Ruixia Guo, Ge Lou, Ziling Liu, Yuanming Shen, Weiwei Feng, Ping Zhang, Jianwei Zhou, Xipeng Wang, Yue Wang, Zhen Shen, Fengxia Xue, Liguo Ma, Chunhua Tu, Yingjie Yang, Wenjun Cheng, Yi Zhang, Shan Kang, Congzhu Li, Hongwu Wen, Xin Wu, Xiping Luo, Rutie Yin, Zhongqiu Lin

**Affiliations:** 1 Department of Gynecologic Oncology, Affiliated Cancer Hospital of Zhengzhou University (Henan Cancer Hospital), Zhengzhou, 450003 China; 2 Department of Gynecological Oncology, Sun Yat-sen Memorial Hospital, Sun Yat-sen University, Guangzhou, 510120 China; 3 Department of Gynecologic Oncology, Shandong Cancer Hospital & Institute, Jinan, 250117 China; 4 Department of Obstetrics and Gynecology, School of Medicine, Zhongda Hospital, Southeast University, Nanjing, 210009 China; 5 Department of Gynecologic Oncology, Fudan University Shanghai Cancer Center, Shanghai, 200032 China; 6 Department of Gynecology, Cancer Hospital Chinese Academy of Medical Sciences, Shenzhen Center, Shenzhen, 518117 China; 7 Department of Gynecology, The Third Affiliated Hospital of Kunming Medical University, Kunming, 650118 China; 8 Department of Obstetrics and Gynecology, Peking Union Medical College Hospital, Beijing, 100730 China; 9 Department of Gynecology, Fourth People’s Hospital of Langfang, Langfang, 065700 China; 10 Department of Obstetrics and Gynecology, Shengjing Hospital of China Medical University, Shenyang, 110022 China; 11 Department of Obstetrics and Gynecology, The First Affiliated Hospital of Xiamen University, Xiamen, 361003 China; 12 Department of Gynecology, The First Affiliated Hospital of Zhengzhou University, Zhengzhou, 450052 China; 13 Department of Gynecology, Harbin Medical University Cancer Hospital, Harbin, 150081 China; 14 Department of Medical Oncology, The First Hospital of Jilin University, Changchun, 130021 China; 15 Department of Gynecologic Oncology, Women’s Hospital School of Medicine, Zhejiang University, Hangzhou, 310006 China; 16 Department of Obstetrics and Gynecology, Ruijin Hospital, Shanghai Jiaotong University School of Medicine, Shanghai, 200025 China; 17 Department of Obstetrics and Gynecology, Zhangjiagang First People’s Hospital, Zhangjiagang, 215600 China; 18 Department of Gynecology, The Second Affiliated Hospital, Zhejiang University School of Medicine, Hangzhou, 310009 China; 19 Department of Obstetrics and Gynecology, Xinhua Hospital, Shanghai Jiao Tong University School of Medicine, Shanghai, 200092 China; 20 Department of Obstetrics and Gynaecology, Henan Provincial People’s Hospital, People’s Hospital of Zhengzhou University, People’s Hospital of Henan University, Zhengzhou, 450003 China; 21 CAS Key Laboratory of Quantum Information, CAS Center for Excellence in Quantum Information and Quantum Physics, Hefei National Laboratory, University of Science and Technology of China, Hefei, 230001 China; 22 Department of Gynecology and Obstetrics, Tianjin Key Laboratory of Female Reproductive Health and Eugenics, Tianjin Medical University General Hospital, Tianjin, 300052 China; 23 Department of Gynecology, Shenzhen People’s Hospital (The Second Clinical Medical College of Jinan University, The First Affiliated Hospital of Southern University of Science and Technology), Shenzhen, 518020 China; 24 Department of Obstetrics and Gynecology, The First Affiliated Hospital of Nanchang University, Nanchang, 330006 China; 25 Department of Surgical Gynecological Tumor, The Affiliated Cancer Hospital of Guizhou Medical University, Guiyang, 550004 China; 26 Department of Gynaecology, The First Affiliated Hospital with Nanjing Medical University, Nanjing, 210029 China; 27 Department of Gynecology, The First Affiliated Hospital of China Medical University, Shenyang, 110001 China; 28 Department of Gynecology, The Fourth Affiliated Hospital of Hebei Medical University, Shijiazhuang, 050011 China; 29 Department of Gynecologic Oncology, Cancer Hospital of Shantou University Medical College, Shantou, 515041 China; 30 Department of Obstetrics and Gynecology, Peking University First Hospital, Beijing, 100034 China; 31 Department of Gynecological Oncology, Obstetrics and Gynecology Hospital of Fudan University, Shanghai, 200011 China; 32 Department of Gynecology and Obstetrics, Guangdong Women and Children Hospital, Guangzhou, 510010 China; 33 Department of Obstetrics and Gynecology, Key Laboratory of Birth Defects and Related Diseases of Women and Children, West China Second University Hospital, Sichuan University, Ministry of Education, Chengdu, 610041 China

**Keywords:** Ovarian cancer, Olaparib, Maintenance therapy, Safety, Chinese patients

## Abstract

**Background:**

Ovarian cancer (OC) is a highly lethal gynecological cancer. Olaparib maintenance therapy was effective and well-tolerated in pivotal RCTs. However, nationwide real-world safety information is limited in China. This multicenter, prospective, observational drug intensive monitoring study monitored the safety of olaparib in a largest-to-date, real-world Chinese OC cohort.

**Methods:**

Eligible OC patients had received ≥ 1 dose of olaparib. Follow-up extended up to 30 days post-olaparib discontinuation or maximally for six months post-enrolment. Primary and secondary endpoints were adverse events (AEs) in all OC patients and in special populations (hepatically/renally impaired before olaparib treatment; aged > 65 years), respectively.

**Results:**

By Jun 30, 2023, 799 patients from 33 sites were enrolled. By data cut-off (Dec 29, 2023), 796 patients treated with olaparib were analyzed. The median age was 55 years (range, 25–85). Of 796 patients, 490 (61.6%) were newly diagnosed and 306 (38.4%) had platinum-sensitive relapsed OC. AEs occurred in 343 (43.1%) patients, and 257 (32.3%) had treatment-related AEs. Anemia (19.2%) was the most common AE. Sixty-eight (8.5%) patients experienced grade ≥ 3 AEs, and 3 had AEs of special interest (AESIs; 0.4%; 1 myelodysplastic syndrome, 1 breast cancer, 1 pneumonitis). 45.2% (19/42) patients with hepatic impairment at baseline, 38.5% (5/13) with renal impairment at baseline and 38.6% (49/127) aged > 65 years experienced any AEs, respectively. No AESIs were reported in these subgroups.

**Conclusions:**

In this largest-to-date, first prospectively enrolled, real-world Chinese OC cohort, olaparib demonstrated a well-tolerated and manageable safety profile (including in special populations) with appropriate management, regardless of treatment lines. No new safety signals were identified.

## Introduction

Ovarian cancer (OC) is the second most lethal gynecological cancer globally, with five-year survival rates of less than 50% and presenting a significant public health challenge [1, 2]. In China, 32,646 deaths from OC were reported in 2022, accounting for 15.8% of the global mortality due to OC [1, 3]. The annual estimate of newly diagnosed OC in China has also been increasing, from 55,342 cases in 2020 to 61,060 cases in 2022 [3, 4]. Such disease burden underscores an urgent need for effective treatment strategies for OC. The standard treatment for OC involving optimal debulking surgery and platinum-based chemotherapy faces the challenge of concerningly high relapse rates reported at 25% in those with early-stage OC and over 70% in those with stage III or IV OC [5–7].

The landscape of OC treatment has been revolutionized by the advent of poly(ADP-ribose) polymerase (PARP) inhibitors. Olaparib, a first-in-class oral PARP inhibitor, has emerged as a guideline-recommended maintenance therapy for OC [8–10], supported by evidence from the pivotal randomized controlled trials (RCTs) of Study 19, SOLO2, SOLO1 and PAOLA-1. In Study 19 (phase 2) and SOLO2 (phase 3), olaparib monotherapy conferred significant progression-free survival benefits over placebo in patients with platinum-sensitive relapsed OC (defined as relapse occurring ≥ 6 months after completing prior platinum-based chemotherapy) who had achieved a complete or partial response (CR or PR) to platinum-based chemotherapy prior to study entry [11, 12]. In SOLO1 (phase 3), olaparib monotherapy significantly reduced the risk of disease progression or death versus placebo in patients with newly diagnosed advanced OC harboring breast cancer gene (*BRCA1*/*BRCA2*) mutations who had achieved a CR or PR to prior platinum-based chemotherapy without the addition of bevacizumab [13]. Lastly in PAOLA-1 (phase 3), olaparib plus bevacizumab showed a significant risk reduction in disease progression or death over bevacizumab alone in patients with newly diagnosed advanced OC who had achieved a CR or PR after first-line platinum-based chemotherapy plus bevacizumab, with greatest benefit seen in patients with homologous recombination deficiency (HRD)-positive tumors (including those without a *BRCA1*/*BRCA2* mutation) [14]. The adverse events (AEs) reported in these RCTs were mostly of grade 1 or 2; the percentage of patients reporting any AEs of grade ≥ 3 was low at 35.3%–39% in the olaparib intervention arm in Study 19, SOLO2 and SOLO1, and was 57% in the olaparib plus bevacizumab arm of PAOLA-1, versus 51% in the placebo plus bevacizumab arm [11–14]. The AEs in these RCTs were often managed by dose reduction or treatment interruption, reflecting a manageable and well-tolerated safety profile of olaparib as monotherapy and in combination with bevacizumab [11–14].

The approval of olaparib in China for OC indications has previously been based on the above mentioned international RCTs: maintenance treatment with olaparib monotherapy was approved in 2018 for platinum-sensitive relapsed OC based on Study 19 and SOLO2, and in 2019 for newly diagnosed, BRCA-mutated OC based on SOLO1; maintenance treatment with olaparib in combination with bevacizumab was approved in 2022 for newly diagnosed advanced, HRD-positive OC based on PAOLA-1. Despite the promising efficacy and safety data from these RCTs, evidence gaps exist regarding the real-world safety profile of olaparib in Chinese OC patients. RCTs typically have stringent inclusion criteria and the patients enrolled therein may not fully represent patients encountered in routine clinical practice [15]. The pivotal RCTs of olaparib in OC excluded patients with abnormal organ function and tended to focus only on the serous and endometrioid subtypes of OC [11–14]. Although some real-world studies have been conducted in China to investigate the use of olaparib in its various approved cancer indications (of which most studies involved patients with OC [16–20]), these were mostly retrospective, single-center studies with limited sample sizes ranging from a few dozen to one or two hundred [16–21].

To establish a more comprehensive understanding of the safety profile of olaparib, a non-interventional drug intensive monitoring (DIM) study was prospectively conducted to monitor the real-world safety of olaparib in the largest Chinese population with approved tumor types. Here, we present the safety and tolerability results of olaparib in the OC patient cohort from this DIM study.

## Methods

### Study design and participants

This DIM study is a multicenter, prospective, observational study (NCT04560452) comprising an OC patient cohort and a prostate cancer (PC) patient cohort. This article only reports on the OC patient cohort (DIM-OC). Eligible OC patients were those who had received ≥ 1 dose of olaparib before enrolment in real-world clinical practice. These patients were consecutively enrolled from 33 participating hospitals across China from March 31, 2021, to June 30, 2023.

The protocol and any amendments thereof were reviewed and approved by the Ethics Committees of participating sites (2020-KY-039). The study was conducted in accordance with the Declaration of Helsinki, Good Clinical Practice, Good Pharmacoepidemiology Practice and the national regulations and guidelines governing local medical practice and ethics. The study information and data in this article are reported in accordance with the Strengthening the Reporting of Observational Studies in Epidemiology (STROBE) statement. All patients provided written informed consent prior to enrolment and patient information was kept under relevant data protection and privacy legislation.

### Study procedures and outcomes

Olaparib was administered at the discretion of the investigators regarding treatment dose and duration. Dose adjustment, interruption or discontinuation were allowed based on investigators’ decisions. Enrolled OC patients were followed up as per the standard clinical practice at the participating sites, until up to 30 days after olaparib discontinuation or for a maximum of six months after enrolment, whichever occurred first. An on-site follow-up was required at least every eight weeks to prevent missing data.

Primary endpoints were the incidence, seriousness, causality, severity and action taken (dose reduction or treatment interruption/discontinuation) of all AEs in all participating OC patients, including AEs of special interest (AESIs, namely, myelodysplastic syndrome [MDS]/acute myeloid leukemia [AML], new primary malignancy other than MDS/AML, and pneumonitis). Secondary endpoints were the incidences of all AEs and AESIs in special populations, including patients with hepatic impairment (Child-Pugh score ≥ 5 points) before olaparib treatment, patients with renal impairment (creatinine clearance [Cockcroft-Gault equation] ≤ 80 mL/min) before olaparib treatment and elderly patients aged > 65 years. Patients were stratified into subgroups for the analysis of secondary endpoints, with the subgroups defined by the presence of hepatic but not renal impairment before olaparib treatment (yes vs. no), the presence of renal but not hepatic impairment before olaparib treatment (yes vs. no), the presence of both hepatic and renal impairment before olaparib treatment (yes vs. no), and age (≤ 65 years vs. >65–70 years vs. >70 years).

Data were collected by investigators using patient medical records, starting from study enrolment and throughout the follow-up period. Data cut-off date was defined as the date six months after the last patient was enrolled. AEs were summarized by preferred terms as per Medical Dictionary for Regulatory Activities v23.0 or a higher version and were graded by Common Terminology Criteria for Adverse Events v5.0.

### Statistical analysis

This DIM study, with its OC and PC cohorts, planned to enroll a total of approximately 1,000 patients, which would yield Clopper-Pearson precision estimates of around 0.7% for 1% incidence of AEs, and around 3.1% for 50% incidence of AEs. For the DIM-OC cohort, approximately 800 patients meeting the inclusion criteria in clinical practice would be enrolled. The full analysis set (FAS) for the DIM-OC cohort consisted of all OC patients who received ≥ 1 dose of olaparib, and was the analysis set for all analyses. Baseline demographic and clinical characteristics were summarized using descriptive statistics. The numbers and percentages of patients with AEs were reported and missing data were not imputed (missing = exclusion). All statistical analyses were performed using SAS v9.4 or a higher version (SAS Institute Inc., Cary, NC, USA).

## Results

### Patient disposition and baseline characteristics

**Fig. 1:**
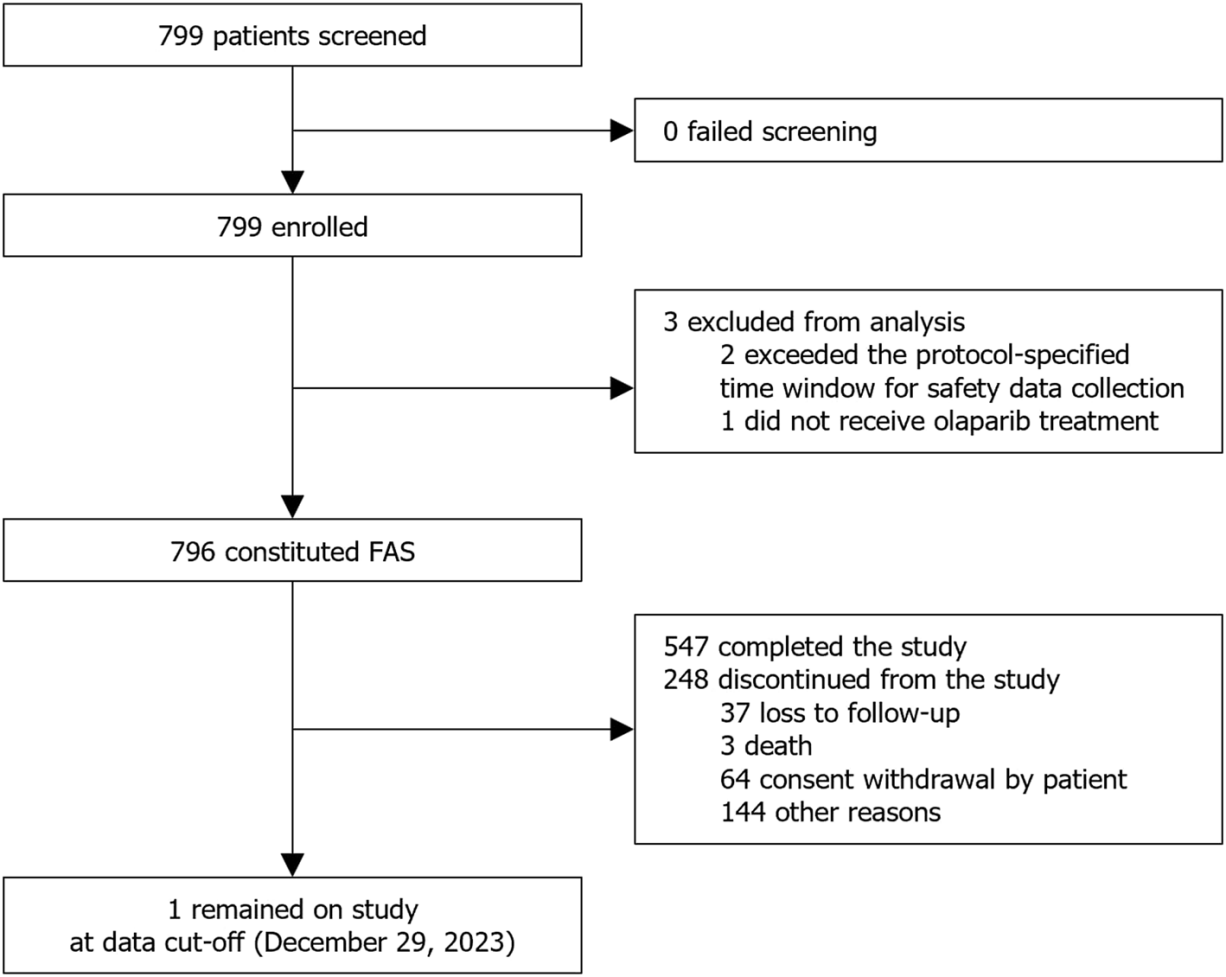
Patient disposition. FAS, full analysis set

Between March 31, 2021, and June 30, 2023, 799 patients were screened and enrolled. The FAS comprised 796 patients, as two of the enrolled patients exceeded the protocol-specified time window for safety data collection and one other patient did not receive olaparib (Fig. 1). By data cut-off (December 29, 2023), 126 (15.8%) patients in the FAS had discontinued olaparib treatment. At enrolment, the median intended olaparib exposure was 4.88 (1.410–12.945) months. Median (interquartile range) time of actual olaparib exposure from the earliest start date of treatment (including treatment received prior to enrolment on study) was 349.0 (244.0–588.0) days.

Patient demographic and clinical characteristics at baseline are presented in Table 1. Median (range) age was 55 (25–85) years, and 79 (9.9%) and 48 (6.0%) patients were aged > 65–70 and > 70 years, respectively. Fifty-two (6.5%) and 23 (2.9%) patients had hepatic and renal impairment before olaparib treatment, respectively, with all impairment being mild or moderate. 490 (61.6%) and 306 (38.4%) patients had newly diagnosed and platinum-sensitive relapsed OC, respectively. The International Federation of Gynecology and Obstetrics (FIGO) staging results at diagnosis were available in 774 patients, most of whom had stage III/IV diseases (648 [83.7%]). BRCA (*BRCA1* and/or *BRCA2*) mutation status (as retrieved from patients’ electronic medical records upon study enrolment) was available in 476 patients, among whom 418 (87.8%) harbored BRCA mutations. Of the 791 patients with prior systemic anticancer therapy, 519 (65.6%) had received only first-line treatment, while 85 (10.7%) had received third-line treatment or beyond.

**Table 1:** Baseline demographic and clinical characteristics

Characteristic, *n* (%)	FAS (*N* = 796)
Median age (range), years	55 (25–85)
Age group	
≤ 65 years	669 (84.0)
> 65–70 years	79 (9.9)
> 70 years	48 (6.0)
With hepatic impairment^a^ before olaparib treatment	52 (6.5)
With renal impairment^b^ before olaparib treatment	23 (2.9)
With both hepatic^a^ and renal^b^ impairments before olaparib treatment	10 (1.3)
Tumor status	
Newly diagnosed	490 (61.6)
Platinum-sensitive relapsed	306 (38.4)
Primary tumor location	
Ovary	701 (88.1)
Fallopian tube	79 (9.9)
Peritoneum	10 (1.3)
Other	6 (0.8)
FIGO stage at diagnosis	*n* = 774^**c**^
I	44 (5.7)
II	82 (10.6)
III	463 (59.8)
IV	185 (23.9)
Histology type	*n* = 793^**d**^
High-grade serous carcinoma	685 (86.4)
Low-grade serous carcinoma	35 (4.4)
Endometrioid	15 (1.9)
Clear cell carcinoma	4 (0.5)
Mucinous adenocarcinoma	28 (3.5)
Germinoma	2 (0.3)
Sarcomatoid carcinoma	1 (0.1)
Other	23 (2.9)
BRCAm status^e^	*n* = 476^**f**^
Positive	418 (87.8)
gBRCAm	247 (51.9)
sBRCAm	56 (11.8)
gBRCAm + sBRCAm	15 (3.2)
tBRCAm with unknown origin^g^	100 (21.0)
Negative	58 (12.2)
Received prior systemic anticancer therapy^h^	*n* = 791
Up to first line	519 (65.6)
Up to second line	187 (23.6)
≥ third line	85 (10.7)

Data were reported based on the principle of “Missing=Exclusion”

*BRCAm* breast cancer gene mutation, *CrCl *creatinine clearance, *FAS *full analysis set, *FIGO *The International Federation of Gynecology and Obstetrics, *gBRCAm *germline BRCA mutation, *sBRCAm *somatic BRCA mutation, *tBRCAm* tumor BRCA mutation

^a^Defined based on Child-Pugh classification system and categorized into mild (Child-Pugh A; scoring 5 to 6 points), moderate (Child-Pugh B; scoring 7 to 9 points) or severe (Child-Pugh C; scoring 10 to 15 points) hepatic impairment

^b^Defined based on CrCl values estimated using the Cockcroft-Gault equation and categorized into mild (CrCl 51–80 mL/min), moderate (CrCl 31–50 mL/min) or severe (CrCl <30 mL/min) renal impairment

^c^Missing data in 22 patients

^d^Missing data in three patients

^e^BRCAm refers to *BRCA1* and/or *BRCA2* mutation. BRCAm status was retrieved from the electronic medical records of the patients upon study enrolment

^f^Based on medical records, BRCAm status had previously been tested in 480 (60.3%) patients, with the test results missing in four patients

^g^Unknown whether it is gBRCAm or sBRCAm

^h^Included cytotoxic chemotherapy (*n*=791 [99.4%]), immunotherapy (*n*=5 [0.6%]), hormonal therapy (*n*=4 [0.5%]), targeted therapy (*n*=208 [26.1%]) and other therapy (*n*=22 [2.8%])

### Safety in the overall OC cohort

In the FAS (*N* = 796), any AEs occurred in 343 (43.1%) patients, and treatment-related AEs (TRAEs) occurred in 257 (32.3%) patients as per investigator assessment. The most common (≥ 5%) any-grade AEs included anemia (*n* = 153 [19.2%]), white blood cell count decreased (*n* = 88 [11.1%]), neutrophil count decreased (*n* = 67 [8.4%]) and platelet count decreased (*n* = 49 [6.2%]; Table 2). Grade ≥ 3 AEs occurred in 68 (8.5%) patients, and grade ≥ 3 TRAEs occurred in 52 (6.5%) patients. The most common (≥ 1%) grade ≥ 3 AEs included anemia (*n* = 43 [5.4%]) and neutrophil count decreased (*n* = 8 [1.0%]; Table 2). Serious AEs occurred in 27 (3.4%) patients. AESIs occurred in 3 (0.4%) patients, with MDS, breast cancer and pneumonitis each occurring in 1 (0.1%) patient; the case of MDS was the only AESI considered treatment-related.

**Table 2:** AEs occurring in ≥ 1% of patients in the overall OC cohort

Patient, *n* (%)	FAS (*N* = 796)	
	Any grade^a^	Grade ≥ 3^b^
Anemia	153 (19.2)	43 (5.4)
White blood cell count decreased	88 (11.1)	7 (0.9)
Neutrophil count decreased	67 (8.4)	8 (1.0)
Platelet count decreased	49 (6.2)	4 (0.5)
Lymphocyte count decreased	27 (3.4)	3 (0.4)
Alanine aminotransferase increased	19 (2.4)	0 (0)
COVID-19	19 (2.4)	0 (0)
Urinary tract infection	19 (2.4)	1 (0.1)
Abnormal hepatic function	17 (2.1)	1 (0.1)
Leukopenia	16 (2.0)	1 (0.1)
Nausea	16 (2.0)	0 (0)
Aspartate aminotransferase increased	14 (1.8)	0 (0)
Blood creatinine increased	14 (1.8)	0 (0)
Decreased appetite	14 (1.8)	0 (0)
Hyperuricemia	14 (1.8)	0 (0)
Myelosuppression	12 (1.5)	3 (0.4)
Vomiting	11 (1.4)	0 (0)
Hypercholesterolemia	8 (1.0)	0 (0)
Insomnia	8 (1.0)	0 (0)
Upper abdominal pain	8 (1.0)	1 (0.1)

AEs were graded according to Common Terminology Criteria for Adverse Events v5.0

*AEs* adverse events, *COVID-19 *coronavirus disease 2019, *FAS *full analysis set

^a^Any-grade AEs with an incidence of at least 1% are listed in descending order of frequency followed by alphabetical order

^b^Zero incidence of grade=5 event for all AEs listed in the table

AEs more often led to treatment interruption (*n* = 43 [5.4%]), rather than dose reduction (*n* = 26 [3.3%]) or treatment discontinuation (*n* = 21 [2.6%]). TRAEs more often led to treatment interruption (*n* = 28 [3.5%]) and dose reduction (*n* = 24 [3.0%]), rather than treatment discontinuation (*n* = 16 [2.0%]). Anemia was the most common TRAE leading to dose reduction (*n* = 12 [1.5%]), treatment interruption (*n* = 18 [2.3%]) and treatment discontinuation (*n* = 6 [0.8%]). Treatment-related death occurred in 1 (0.1%) patient and was due to MDS. No new safety signals were identified in the overall OC cohort.

### Safety in special populations

Incidences of any AEs in the special populations were largely comparable with those in the respective comparator subgroups, except for the numerically higher incidence in the subgroup with both hepatic and renal impairment before olaparib treatment (Table 3). The three patients who experienced AESIs were all aged ≤ 65 years. One patient who experienced an AESI of MDS had both hepatic and renal impairment before olaparib treatment, while the other two patients who experienced AESIs had neither impairment (Table 3). It should be noted that the follow-up duration in the DIM-OC study was only six months, which is insufficient for assessing long-term hematological toxicities such as MDS/AML. Anemia was the most common AE in patients with hepatic but not renal impairment (7/42 [16.7%]), with renal but not hepatic impairment (3/13 [23.1%]), and with both renal and hepatic impairment (3/10 [30.0%]) before olaparib treatment. The most common (≥ 3%) AEs in patients aged > 65 years included anemia (20/127 [15.7%]), neutrophil count decreased (6/127 [4.7%]), COVID-19 (6/127 [4.7%]), white blood cell count decreased (5/127 [3.9%]), and blood creatinine increased (5/127 [3.9%]).

**Table 3:** Any AEs and AESIs in special populations

Special population, *n* (%)	Any AEs	AESIs
With hepatic but not renal impairment before olaparib treatment		
Yes (*n* = 42)	19 (45.2)	0 (0)
No (*n* = 754)	324 (43.0)	3 (0.4)
With renal but not hepatic impairment before olaparib treatment		
Yes (*n* = 13)	5 (38.5)	0 (0)
No (*n* = 783)	338 (43.2)	3 (0.4)
With both hepatic and renal impairment before olaparib treatment		
Yes (*n* = 10)	6 (60.0)	1 (10.0)
No (*n* = 786)	337 (42.9)	2 (0.3)
Age		
≤ 65 years (*n* = 669)	294 (43.9)	3 (0.4)
> 65–70 years (*n* = 79)	31 (39.2)	0 (0)
> 70 years (*n* = 48)	18 (37.5)	0 (0)

AEs were graded according to Common Terminology Criteria for Adverse Events v5.0

*AEs* adverse events, *AESIs *adverse events of special interest

## Discussion

To our knowledge, the DIM-OC cohort was the first prospectively enrolled Chinese patient cohort to evaluate the real-world safety of olaparib in OC. It fills critical evidence gaps in the study of olaparib by providing safety evidence from a diverse patient population representative of those encountered in routine care, with the largest sample size to date. In DIM-OC, olaparib demonstrated a well-tolerated and manageable safety profile across the entire cohort, irrespective of treatment lines. Moreover, the safety profiles observed in special populations, including patients with hepatic or renal impairment prior to olaparib treatment and elderly patients, were generally consistent with that in the overall OC cohort, suggesting potential suitability of olaparib for use in a broad range of OC patients.

The real-world safety and tolerability of olaparib maintenance therapy observed in DIM-OC was consistent with that in the pivotal trials of SOLO1 and SOLO2, and also with the findings from L-MOCA, a single-arm, phase 3 study on platinum-sensitive relapsed OC where over 90% of the patients were recruited from China [12, 13, 22]. In DIM-OC, hematological events emerged as the most common type of AEs, in line with the frequent incidence of such events reported in SOLO2, SOLO1 and L-MOCA [12, 13, 22], as well as reflecting the general safety profile observed with PARP inhibitors [23]. Anemia in particular was the most frequently reported AE across both DIM-OC and the clinical trials. The management of AEs in DIM-OC predominantly involved treatment interruption (5.4%) and dose reduction (3.3%) rather than treatment discontinuation (2.6%), which is in line with the strategies employed in SOLO2 (45%, 25% and 11%, respectively) and SOLO1 (52%, 28% and 12%, respectively) [12, 13]. Together, these findings reaffirm the safety of olaparib in Chinese patients with OC.

The incidences of any-grade anemia and grade 3 or 4 anemia were markedly lower in DIM-OC (19.2% and 5.4%) than in SOLO2 (43.6% and 19.5%), SOLO1 (39% and 22%) and L-MOCA (76.3% and 25.0%) [12, 13, 22]. Similar trends were present for AE management, where the frequencies of actions taken to manage AEs (dose reduction, treatment interruption or discontinuation) were numerically lower in DIM-OC than in the clinical trials [12, 13, 22]. When interpreting these results, it is important to note that unlike the clinical trials which exclusively enrolled patients without prior exposure to olaparib [12, 13, 22], DIM-OC enrolled patients by the criterion of “having received ≥ 1 dose of olaparib before enrolment”. The median intended olaparib exposure at enrolment was 4.88 (1.410–12.945) months. Consequently, DIM-OC have included some patients who had already exhibited or developed the ability to tolerate prolonged olaparib treatment, and/or some patients who had undertaken AE management, such as dose reduction, prior to study entry. Such patients would likely have fewer AEs and less need for AE management during the data collection period of DIM-OC, thereby contributing to the lower AE incidences and the less frequent actions for AE management. A retrospective real-world study on PARP inhibitors in platinum-sensitive recurrent OC also reported numerically lower AE incidences than in clinical trials [16]: in the 54 patients enrolled who had been receiving olaparib (with records of dose reduction in 21 patients and dose interruption in 13 at baseline), the incidences reported were 34.7% for any-grade anemia, and 10.7% for grade ≥ 3 anemia [16]. Unlike clinical trials, real-world studies could lack proactive monitoring for AEs. The reliance on spontaneous reporting and routine medical records inherently leads to an underestimation of the true incidence of AEs. Despite the possible bias caused by including patients with prior exposure, the low levels of AE incidence observed in DIM-OC likely still reflect the real-world situation where patients can receive olaparib treatment with an acceptable safety and tolerability profile with appropriate AE management.

Olaparib, being a small-molecule targeted agent, is metabolized primarily via the liver and the kidney [24, 25]. Therefore, hepatic and renal impairment could possibly affect its pharmacokinetics (PK) and lead to toxicity not expected in individuals with normal hepatic and renal function. Early clinical trials of olaparib, such as SOLO2 and SOLO1, included patients with normal or mildly abnormal organ functions [12, 13]. Subsequent studies further explored the association of olaparib toxicity with kidney or liver dysfunction. One study reported markedly elevated olaparib exposure in patients with moderate renal impairment and warned about the risk of increased toxicity and AE incidence in such patients without dose adjustment [26]. Conversely, a study in hepatically impaired patients found neither significant changes in olaparib exposure nor major concerns regarding increased AE incidence for patients with mild or moderate impairment [27]. DIM-OC endeavored to add to the evidence base regarding the safety of olaparib in patients with renal and/or hepatic impairment before olaparib treatment, despite the limited subgroup sample sizes (*n* < 50). As discussed above, the inclusion of patients with prior olaparib exposure might have led to an underestimation of the frequencies of AEs and of AE management actions. Nevertheless, the general consistency in safety results between these subgroups and the overall DIM-OC cohort preliminarily suggests an acceptable safety profile of olaparib in these special populations, where the potential elevated toxicity of olaparib could possibly be avoided or resolved through appropriate AE management.

There are limitations in this study. Firstly, DIM-OC had a relatively short follow-up duration of a maximum of 6 months, limiting the capture of longer-term safety information of olaparib, especially for the occurrence of MDS/AML which tends to emerge after prolonged treatment [28]. Secondly, the study was conducted during the COVID-19 pandemic, when it was difficult or unfeasible for some patients to attend follow-up visits. This situation was compounded by the non-interventional nature of this real-world study, where the protocol-specified on-site follow-up at least once every eight weeks was not mandatory. As such, some AEs had probably been missed in data collection. Thirdly, some patients had already started olaparib treatment before study entry, for whom the occurrence and management of AEs prior to study enrolment could not be captured. Nevertheless, since this study aimed to collect as much real-world data as possible by being all-encompassing in patient recruitment, those patients with prior olaparib exposure had not been excluded. Additionally, BRCA mutation status was obtained from electronic medical records at enrolment, which revealed approximately 40% missing data. This may constitute a further limitation of the study. Finally, no data were available for patients with severe hepatic or renal impairment before olaparib treatment, as there were no such patients in the special populations investigated. A simulation study using physiologically based PK data predicted that the area under the curve (AUC) of olaparib tablet could increase by 121% and 288% in patients with severe renal or hepatic impairment, respectively, suggesting the potential concern of elevated toxicity [29]. Given the absence of clinical trial data for olaparib in these patient populations, physicians are unlikely to prescribe olaparib to such patients in clinical practice, thereby precluding the collection of real-world evidence. Further research would be needed to gather safety information and inform treatment decisions for these patient populations.

## Conclusion

In conclusion, the DIM-OC study reaffirmed the acceptable and well-tolerated safety profile of olaparib across treatment lines when appropriate supportive care was provided in this largest-to-date, first prospectively enrolled, real-world Chinese OC cohort. Results were consistent with pivotal RCTs, and no new safety signals were detected. The consistent safety profile of olaparib in the special populations compared with the overall OC cohort preliminarily supports its use in these patient populations, for whom further efficacy and safety verification is needed to better inform clinical practice.

## Data Availability

Data underlying the findings described in this manuscript are available from the corresponding authors upon reasonable request, in accordance with AstraZeneca’s data sharing policy.

## Abbreviations

AE: Adverse event
AESI: Adverse event of special interest
AML: Acute myeloid leukemia
AUC: Area under the curve
BRCA: Breast cancer gene
BRCAm: Breast cancer gene mutation
COVID-19: Coronavirus disease 2019
CR: Complete response
CrCl: Creatinine clearance
DIM: Drug intensive monitoring
FAS: Full analysis set
FIGO: The International Federation of Gynecology and Obstetrics
gBRCAm: Germline breast cancer gene mutation
HRD: Homologous recombination deficiency
MDS: Myelodysplastic syndrome
OC: Ovarian cancer
PARP: Poly(ADP-ribose) polymerase
PC: Prostate cancer
PK: Pharmacokinetics
PR: Partial response
RCT: Randomized controlled trial
sBRCAm: Somatic breast cancer gene mutation
STROBE: Strengthening the Reporting of Observational Studies in Epidemiology
TRAE: Treatment-related adverse event
tBRCAm: Tumor breast cancer gene mutation
